# Quality of Life and Gluten-Free Diet Adherence in Adult Patients with Celiac Disease: A Cross-Sectional Questionnaire Study

**DOI:** 10.3390/nu18162701

**Published:** 2026-08-19

**Authors:** Zuzanna Zając, Aleksandra Kołtuniuk

**Affiliations:** Department of Nursing, Faculty of Nursing and Midwifery, Wroclaw Medical University, 51-618 Wrocław, Poland

**Keywords:** celiac disease, gluten-free diet, quality of life, dietary adherence, GIQLI, CDAT, chronic disease, health education

## Abstract

***Background:*** Celiac disease is a chronic autoimmune enteropathy whose only established treatment is a strict, lifelong gluten-free diet (GFD). Beyond physical symptoms, the disease and its dietary management affect emotional, social, and economic functioning. ***Objectives:*** This study aimed to assess health-related quality of life (HRQoL) in adult patients with celiac disease and to identify factors associated with their daily functioning and adherence to the GFD. ***Methods:*** A cross-sectional survey was conducted between February and March 2026 among 101 adults with a self-reported diagnosis of celiac disease made by a gastroenterologist, recruited through online support groups. Data were collected using an author-designed sociodemographic questionnaire, the Gastrointestinal Quality of Life Index (GIQLI), and the Celiac Dietary Adherence Test (CDAT). Analyses (Mann–Whitney U and Kruskal–Wallis tests, repeated-measures ANOVA, Spearman correlations with false-discovery-rate correction, and multivariable linear and logistic regression with prespecified sensitivity analyses) were performed in R 4.5.2, with α = 0.05. ***Results:*** The most frequently reported difficulties were eating away from home, the high cost of the GFD, and limited product availability; the most common symptoms after inadvertent gluten exposure were abdominal pain, bloating, fatigue, diarrhea, and headache. HRQoL was lowest in the emotional and physical domains. Better dietary adherence was significantly correlated with higher HRQoL across the total score and all domains except the social domain, whose weak internal consistency in this sample (α = 0.57) precludes substantive interpretation (total GIQLI: ρ = −0.50; *p* < 0.001), and in the multiple regression, adherence remained the strongest independent correlate of HRQoL (β = −0.36; adjusted R^2^ = 0.41). Longer time on the diet was independently associated with better adherence (adjusted OR = 0.55 per duration category; *p* = 0.017). In a small vocational education subgroup (*n* = 8), poorer adherence and lower HRQoL were observed relative to participants with higher education, though only the HRQoL difference survived correction for multiple testing (FDR-adjusted *p* = 0.011 vs. 0.060 for adherence); given the size of this subgroup, this comparison is exploratory and hypothesis-generating. Higher HRQoL was also associated with milder symptoms, better-rated medical care, greater product availability, and higher household income. ***Conclusions***: In this sample, HRQoL scores were lowest in the emotional and physical domains; because the design was cross-sectional and included no comparison group, these data characterize the distribution of HRQoL within the sample and do not establish that celiac disease reduces HRQoL. Dietary adherence was the strongest correlate of patient functioning, alongside symptom burden, self-rated quality of medical care, and product availability. Because the design is cross-sectional, these associations do not establish direction, and whether comprehensive health education and psychological support improve outcomes requires prospective and interventional evaluation.

## 1. Introduction

Celiac disease, also known as coeliac disease, is a chronic, multisystem autoimmune disorder triggered by dietary gluten in genetically predisposed individuals carrying the HLA-DQ2 and/or HLA-DQ8 haplotypes [1,2]. Ingestion of gluten (a protein found in wheat, rye, and barley) leads to damage to the small-intestinal mucosa, villous atrophy, and malabsorption of nutrients [3,4,5]. Over recent decades, the number of diagnosed cases has increased, reflecting both greater public and clinical awareness and improvements in serological diagnostics [6,7,8]. The disease may present at any age, and its clinical picture is highly heterogeneous, ranging from classic gastrointestinal symptoms to extraintestinal manifestations such as anemia, osteoporosis, and neurological or psychiatric disorders [1,9,10,11].

Current epidemiological data indicate that celiac disease affects, on average, approximately 1.4% of the global population serologically and 0.7% based on biopsy confirmation, and is more frequent in women than in men [12,13,14]. In Poland, the true prevalence is difficult to establish because no national registry exists; pediatric prevalence has been estimated at 1:404, suggesting substantial underdiagnosis compared with high-detection countries such as Finland (1:50) or Sweden (1:190), a phenomenon often described as a “statistical iceberg” [15,16,17]. Diagnostic delay remains common. In Poland, the mean time from first symptoms to diagnosis has been estimated at around seven years, and up to nine years in adults [18].

According to current guidelines from the American College of Gastroenterology, the British Society of Gastroenterology, and the European Society for the Study of Coeliac Disease, the cornerstone of treatment remains a strict, lifelong gluten-free diet (GFD) [1,9,19,20,21]. Adherence to dietary recommendations promotes mucosal recovery and reduces the risk of complications, including nutritional deficiencies, osteoporosis, neurological disorders, and small-bowel lymphoma [22,23]. At the same time, the GFD is associated with considerable economic, social, and psychological burden. Gluten-free products are frequently more expensive and less available than their conventional counterparts, imposing a financial strain on patients and their families [24].

Because treatment relies on a permanent lifestyle change rather than pharmacotherapy, celiac disease is a particularly informative context in which to study health-related quality of life (HRQoL). The World Health Organization defines quality of life as an individual’s perception of their position in life within their cultural and value context; HRQoL narrows this down to the physical, psychological, and social domains directly connected to health [25,26]. In celiac disease, the diet affects all of these domains, from resolution of physical symptoms and mucosal regeneration, to the ongoing anxiety of accidental gluten exposure and a sense of being different, to social difficulties surrounding eating outside the home, travel restrictions, and the high cost of specialized foods [27,28].

Given the chronic nature of the disease and its multidimensional impact on physical, emotional, and social functioning, the quality of life of patients with celiac disease is an important issue for contemporary clinical and nursing care. Analyzing the factors that shape patient well-being helps to quantify the everyday burden of the disease and to develop effective support strategies that reach beyond dietary counseling. The present study, therefore, aimed to assess HRQoL in adult patients with celiac disease and to identify sociodemographic and clinical factors associated with their daily functioning and adherence to the gluten-free diet.

Although the relationship between gluten-free diet adherence and quality of life has been examined in several populations, three gaps remain that the present study addresses. First, data from adult Polish patients collected with standardized instruments are limited, despite documented underdiagnosis and long diagnostic delay in this country. Second, the substantial item overlap between adherence measures and gastrointestinal quality-of-life instruments is rarely examined, so it is generally unclear how much of the reported association is attributable to shared symptom content. Third, modifiable contextual factors—perceived stigmatization, self-rated quality of medical care, and the local availability of gluten-free products—are seldom modeled together with adherence. The present study, therefore, aims not only to describe the adherence–quality of life association but also to test its robustness to measurement overlap and to place it alongside these contextual correlates.

## 2. Materials and Methods

### 2.1. Study Design and Participants

This cross-sectional study used a diagnostic survey method with a questionnaire technique. Data were collected between February and March 2026. Participants were recruited through online support groups and forums for people with celiac disease and those following a gluten-free diet, allowing enrollment of respondents from across Poland. Recruitment was based on a non-probability, self-selected (convenience) sample. An invitation containing study information and a link to an anonymous online questionnaire was posted in Polish-language internet support groups and discussion forums dedicated to celiac disease and to the gluten-free diet. Participation was voluntary, anonymous and unpaid, and no incentive was offered.

The study group comprised 101 adults with celiac disease. The inclusion criteria were age ≥ 18 years, a diagnosis of celiac disease made by a gastroenterologist, and current use of a gluten-free diet. The exclusion criteria were absence of a formal diagnosis, lack of consent to participate, and the presence of mental disorders or intellectual disability that could preclude reliable completion and comprehension of the questionnaire. Respondents were informed that the collected information would be used solely for scientific purposes; submission of a completed form was considered equivalent to informed consent to participate. Of the 104 questionnaires submitted, three were excluded because the respondents were under 18 years of age; the remaining 101 met all eligibility criteria and were analyzed. Eligibility with respect to diagnosis was based on the respondent’s own declaration that celiac disease had been diagnosed by a gastroenterologist; respondents were not asked to indicate how the diagnosis had been established (serology, duodenal biopsy, or both), and no serological, histological or medical record verification was performed. All diagnoses reported here are, therefore, self-reported gastroenterologist-made diagnoses.

### 2.2. Instruments

#### 2.2.1. Author-Designed Questionnaire

The author-designed questionnaire complemented the standardized instruments and comprised 15 items (closed, multiple-choice, and open-ended). It captured the sociodemographic profile of the group (sex, age, education, and place of residence by settlement size), household characteristics (net household income, household size, and average monthly food expenditure), and disease- and diet-related variables, including duration of the gluten-free diet, self-rated quality of medical care in Poland, bodily reactions to accidental gluten exposure, availability of gluten-free products in the respondent’s area, and everyday difficulties such as eating outside the home. A key item addressed perceived stigmatization or discrimination due to the disease in public settings and at work, directly relevant to the social dimension of quality of life.

The questionnaire was developed by the authors for the purposes of this study. Items were generated on the basis of a review of the literature on the burden of the gluten-free diet [24,27,28] and of clinical experience in the care of adults with celiac disease. Before administration, the draft questionnaire was reviewed for content relevance, clarity and completeness by one specialist dietitian experienced in the care of adults with celiac disease, and the comments obtained were incorporated into the final wording; no formal pilot reliability study or psychometric validation was undertaken. The questionnaire is not a validated psychometric scale, and its items were analyzed individually rather than as a composite score; no reliability coefficient is, therefore, reported for it. The full item wording, with all response options as presented to respondents and the coding applied in the analyses, is provided in Supplementary Material S1.

Reactions to inadvertent gluten exposure were captured by two items. The first was a multiple-response checklist of symptoms (abdominal pain, bloating, diarrhea, headache, fatigue/weakness, concentration problems, constipation, nausea/vomiting, joint pain, skin rash, and other), respondents could indicate more than one symptom. The second was a single ordinal item asking how often those symptoms occur after consuming gluten-containing products, with the response options Never, Rarely, Sometimes, Often, Always and Not applicable, coded 0, 1, 2, 3, 4 and 0 respectively; “Not applicable” and “Never” share the lowest code because the former option was offered only to respondents who had already indicated that they experience no symptoms after gluten exposure. This second item was entered as a ranked score in the correlation and regression models, and it is referred to throughout as symptom frequency, since it measures how often post-exposure symptoms occur rather than how intense they are.

#### 2.2.2. Gastrointestinal Quality of Life Index (GIQLI)

The Gastrointestinal Quality of Life Index (GIQLI), developed by Eypasch et al. [29], is a validated tool for assessing quality of life in patients with gastrointestinal disorders. It comprises 36 items grouped into five domains: symptoms (19 items), emotions (5 items), physical function (7 items), social function (4 items), and medical treatment (1 item). Each item is scored from 0 to 4, giving a maximum total of 144 points, where a higher score indicates better quality of life. The GIQLI is recommended by the European Study Group for Antireflux Surgery (ESGARS), which supports its reliability for assessing HRQoL in gastrointestinal disease. Although the GIQLI is a generic gastrointestinal instrument rather than a celiac-specific measure, its symptom, emotional, physical, and social domains capture burdens central to living with a gluten-free diet. The Polish version used here is an item-by-item, order-preserving translation of the original 36-item instrument; we did not identify a separately published psychometric validation or domain key for a Polish adaptation, and domain scores were, therefore, computed using the domain structure of the original GIQLI-36 (Eypasch et al. [29]), in which the physical function domain comprises seven items, and the social function domain comprises four. Internal consistency in the present sample was good to excellent for the total score and for all multi-item domains except the social domain (Section 3.7). The GIQLI was chosen because it is widely used in Polish gastroenterological research and, therefore, permits comparison across patient groups and because celiac-specific instruments such as the CD-QOL or CDQ lacked a validated Polish adaptation at the time of data collection.

#### 2.2.3. Celiac Dietary Adherence Test (CDAT)

The Celiac Dietary Adherence Test (CDAT), developed by Leffler et al. in 2009 [30], is a self-assessment tool measuring adherence to the gluten-free diet and identifying patients at risk of inadvertent or intentional gluten intake. It comprises 7 items addressing clinical symptoms, patient awareness of health consequences, and determination to maintain the dietary regimen. Each item is scored from 1 to 5, yielding a total from 7 to 35 points, where a score of 7–12 points indicates very good adherence, and a score above 17 points indicates insufficient dietary discipline and a high risk of gluten exposure. Importantly, in the CDAT, a lower score reflects better adherence. The test has demonstrated high sensitivity and specificity for identifying non-adherent patients in the Polish population, correlating strongly with professional dietary assessment and serology [31], supporting its use as a rapid and reliable monitoring tool. In the present study, the established cut-off of CDAT ≥ 13 was used to classify inadequate adherence.

### 2.3. Statistical Analysis

Quantitative variables were summarized as mean, median, standard deviation, skewness, kurtosis, minimum, and maximum values; categorical variables were expressed as counts and percentages. The distribution of each quantitative variable was examined with the Shapiro–Wilk test together with skewness and kurtosis. Because the CDAT score and the total GIQLI score departed from normality (CDAT: Shapiro–Wilk W = 0.93 (*p* < 0.001); skewness = 1.16), non-parametric methods (Spearman’s ρ, Mann–Whitney U, and Kruskal–Wallis) were used as the primary tests; parametric tests were used only where their assumptions were satisfied, and where both were applied, the results of each are reported. Effect estimates are reported with 95% confidence intervals where these are available.

The internal consistency of the GIQLI (total score and the five domain subscales) and of the CDAT was assessed in the present sample using Cronbach’s α, with α ≥ 0.70 considered acceptable (α coefficients are reported in Section 3.7).

Between-group comparisons used the Mann–Whitney U test for two groups and the Kruskal–Wallis test for the three education categories, with Dunn’s post hoc test and Bonferroni adjustment. Each comparison is reported with an effect size: the rank-biserial correlation for two-group comparisons and epsilon-squared, computed as H/(*n* − 1), for the Kruskal–Wallis tests. The omnibus group comparisons were corrected for multiple testing separately from the correlation analyses and formed a single Benjamini–Hochberg family of 41 tests: the seven outcomes (the CDAT score, total GIQLI score and five GIQLI domains) compared across the six grouping variables (sex, education, diet duration, gluten-free product availability, self-rated medical care, and adherence status), except for the CDAT score across adherence status, which that variable defines. For each outcome, the primary test was chosen on the basis of its distribution, so this family contained Mann–Whitney and Kruskal–Wallis *p*-values for the non-normally distributed outcomes and *t*-test and ANOVA *p*-values where the parametric assumptions were met; eight of the 41 comparisons remained significant after adjustment, and both unadjusted and FDR-adjusted *p*-values are reported (Section 3.3 and Section 3.4). Dunn’s pairwise post hoc contrasts were Bonferroni-adjusted within the corresponding omnibus test only, so that no comparison received two corrections. Education was additionally coded as a ranked ordinal score (vocational < secondary < higher) for the multivariable models; because this coding assumes an approximately linear ordering of educational attainment, it was checked in a sensitivity analysis in which education was entered as a categorical predictor.

Differences among the five GIQLI domains (expressed as mean item scores) were examined with a repeated-measures ANOVA; sphericity was evaluated with Mauchly’s test, and because it was violated (Mauchly’s W = 0.407; *p* < 0.001), the Greenhouse–Geisser correction was applied, with the Friedman test used as a non-parametric check. Pairwise post hoc comparisons between domains used Wilcoxon signed-rank tests with Bonferroni adjustment across the ten domain pairs. Because the medical treatment domain comprised a single item, any cross-domain comparison involving it was interpreted with caution.

Bivariate associations were quantified with Spearman’s ρ. Given the number of correlations tested, *p*-values were adjusted using the Benjamini–Hochberg false-discovery-rate (FDR) procedure, and both unadjusted and adjusted values were reported. The main correlation family comprised the 78 pairwise associations among the GIQLI scores, the CDAT score and the study covariates. Two further sets of tests were treated as separate families: the five correlations between perceived stigmatization and the individual GIQLI domains (Section 3.5), and the six correlations involving household income and food expenditure (Section 3.10).

Because the CDAT and the GIQLI symptom domain share symptom-related item content, the adherence–quality of life association was additionally examined (i) after excluding the GIQLI symptom domain and (ii) with partial correlation controlling for post-exposure symptom frequency, to test whether the association persisted independently of shared symptom content.

Two multivariable models were used to identify factors independently associated with the outcomes. A multiple linear regression modeled the total GIQLI score as a function of dietary adherence (CDAT), education, symptom frequency, self-rated medical care, product availability, diet duration, age, and sex; standardized coefficients (β), 95% CIs, and the adjusted R^2^ are reported. A binary logistic regression modeled inadequate adherence (CDAT ≥ 13, per the standard cut-off [30]) using the same candidate predictors other than the CDAT score itself, which defines the outcome, reporting adjusted odds ratios and 95% CIs. Model assumptions (linearity, multicollinearity via variance inflation factors, and residual diagnostics) were verified; all variance inflation factors ≤ 1.27, with no residual or influential-case concerns. Three sensitivity analyses were performed: (i) both multivariable models were refitted with education entered as a categorical rather than a ranked predictor (with higher education as the reference category); (ii) the logistic model was re-estimated using Firth’s penalized likelihood and, additionally, as a parsimonious model restricted to the predictors that were significant in the full model; and (iii) household income and average monthly food expenditure were examined in relation to both outcomes.

All analyses were performed in R 4.5.2 (R Core Team; packages psych, effectsize, and rstatix); the significance level was set at α = 0.05 (two-tailed).

### 2.4. Ethics

This study was conducted in accordance with the ethical principles of the Declaration of Helsinki of the World Medical Association and was approved by the Bioethics Committee at Wrocław Medical University (approval no. 70/2026). Throughout the survey, priority was given to ensuring participant safety, respecting dignity, and protecting the privacy of all respondents. Participation was voluntary and anonymous, and submission of a completed questionnaire constituted informed consent.

## 3. Results

### 3.1. Characteristics of the Study Group

The study group comprised 101 adults with diagnosed celiac disease. Women constituted the clear majority (84.16%, *n* = 85), with men accounting for 15.84% (*n* = 16). The mean age was 34.37 ± 9.07 years (range: 19–56). Most participants had higher education (70.30%, *n* = 71), followed by secondary (21.78%, *n* = 22) and vocational education (7.92%, *n* = 8). By place of residence, the largest group lived in cities of more than 500,000 inhabitants (39.60%, *n* = 40), followed by rural areas (25.74%, *n* = 26). In most households, the respondent was the only person following a gluten-free diet (77.23%, *n* = 78; this item was left unanswered by one respondent). The full sociodemographic profile is presented in Table 1.

The duration of the gluten-free diet varied: 23.76% of respondents had followed it for less than one year, 30.69% for 1–3 years, 18.81% for 4–10 years, and 26.73% for more than 10 years. The most frequently reported difficulties were eating outside the home (97.03%), the cost of the gluten-free diet (81.19%), and difficulty finding suitable products (58.42%). Fear of inadvertent gluten exposure was reported in the free-text answers entered under the “other” option of the same item. Perceived stigmatization was common; 51.49% of respondents reported experiencing it often and a further 38.61% sometimes. The most frequent symptoms after accidental gluten intake were abdominal pain (73.27%), bloating (68.32%), fatigue/weakness (46.53%), diarrhea (44.55%), and headache (40.59%). Availability of gluten-free products in the respondent’s area—an item distinct from the difficulty checklist above—was rated as always available by 6.93% of respondents (*n* = 7), usually available by 73.27% (*n* = 74), and rarely available by 17.82% (*n* = 18), while 1.98% (*n* = 2) reported that no gluten-free products were available in their area. The social and clinical profile is summarized in Table 2.

### 3.2. Quality of Life (GIQLI) and Dietary Adherence (CDAT)

Descriptive statistics for the quantitative variables are shown in Table 3. The mean total GIQLI score was 94.13 ± 22.80 (range: 31–141), and the mean CDAT score was 15.24 ± 4.40 (range: 7–35).

According to the CDAT, almost half of the respondents (43.56%) achieved an intermediate adherence score; 29.70% achieved the lowest scores, indicating good adherence; and 26.73% obtained scores suggesting a need for additional support. Applying the established clinical cut-off, 70.30% (*n* = 71) were classified as having inadequate adherence (CDAT ≥ 13).

Analysis showed that patients reported the highest quality of life in the social domain (M = 2.98; SD = 0.72), followed by the symptom domain (M = 2.82; SD = 0.70); the medical (M = 2.30; SD = 1.18), physical (M = 2.24; SD = 0.79), and emotional (M = 2.13; SD = 0.74) domains received the lowest scores.

The five GIQLI domains, expressed as mean item scores, differed significantly from one another (repeated-measures ANOVA with Greenhouse–Geisser correction, F(2.59, 259.25) = 42.90, *p* < 0.001, and generalized η^2^ = 0.14; Friedman χ^2^(4) = 126.16 and *p* < 0.001). In Bonferroni-adjusted pairwise post hoc comparisons, the five domains separated into two groups whose members did not differ from one another: the higher-scoring symptom and social domains (symptoms versus social, adjusted *p* = 0.148), and the lower-scoring emotional, physical and medical treatment domains (all adjusted *p* = 1.000). Every comparison across the two groups was significant (all adjusted *p* < 0.001). These data, therefore, support the conclusion that quality of life is lower in the emotional, physical and treatment-related domains than in the symptom and social domains, rather than that any single domain is the most affected. Because the medical treatment domain comprises a single item, comparisons involving it are interpreted with caution.

### 3.3. Dietary Adherence (CDAT) by Sociodemographic Variables

Dietary adherence did not differ significantly between men (*n* = 16; M = 15.56; SD = 5.15) and women (*n* = 85; M = 15.18; SD = 4.28) (*p* = 0.78; FDR-adjusted *p* = 0.82; rank-biserial r = −0.04 (95% CI: −0.34 to 0.26)). Adherence did not correlate with age (ρ = −0.15; FDR-adjusted *p* = 0.20), and its weak correlation with diet duration (ρ = −0.21; unadjusted *p* = 0.036) did not survive correction for multiple testing (FDR-adjusted *p* = 0.062). Adherence did correlate with education (ρ = −0.28; FDR-adjusted *p* = 0.010) and with product availability (ρ = −0.25; FDR-adjusted *p* = 0.022), in both cases, in the direction of better adherence. In the group comparison, adherence differed across education categories at the uncorrected level only (Kruskal–Wallis H(2) = 8.20, unadjusted *p* = 0.017, FDR-adjusted *p* = 0.060, and ε^2^ = 0.08, a small-to-moderate effect). Participants with vocational education (*n* = 8; M = 18.38; SD = 4.84) had the highest CDAT scores, indicating the poorest adherence, followed by those with secondary (*n* = 22; M = 16.27; SD = 3.64) and higher education (*n* = 71; M = 14.56; SD = 4.41), although no pairwise contrast reached significance after Bonferroni adjustment (vocational versus higher education, adjusted *p* = 0.063).

### 3.4. Quality of Life (GIQLI) by Sociodemographic Variables

Overall quality of life did not differ significantly between men (*n* = 16; M = 100.63; SD = 25.36) and women (*n* = 85; M = 92.91; SD = 22.23): Mann–Whitney *p* = 0.149 (FDR-adjusted *p* = 0.235; Student’s t(99) = 1.25 (*p* = 0.216)) and rank-biserial r = −0.23 (95% CI: −0.50 to 0.08). Quality of life correlated with neither age (ρ = −0.04; FDR-adjusted *p* = 0.80) nor diet duration (ρ = 0.15; FDR-adjusted *p* = 0.21), but did correlate with education (ρ = 0.32; FDR-adjusted *p* = 0.003). Education was also associated with quality of life in the group comparison (Kruskal–Wallis H(2) = 12.60, unadjusted *p* = 0.002, FDR-adjusted *p* = 0.011, and ε^2^ = 0.13, a small-to-moderate effect). Participants with vocational education (*n* = 8; M = 67.13; SD = 23.33) reported a lower quality of life than those with higher education (*n* = 71; M = 98.80; SD = 20.96; Bonferroni-adjusted *p* = 0.003), whereas neither the contrast with secondary education (*n* = 22; M = 88.86; SD = 21.16; adjusted *p* = 0.148) nor that between secondary and higher education (adjusted *p* = 0.240) was significant. Because the vocational-education subgroup comprised only eight participants, its mean was estimated imprecisely (standard error of approximately 8.2 GIQLI points), and this comparison should be regarded as exploratory and hypothesis-generating rather than confirmatory.

### 3.5. Correlates of Quality of Life: Stigmatization, Symptom Frequency, Medical Care, and Product Availability

A series of Spearman correlation analyses (Table 4) examined the associations between overall quality of life and perceived stigmatization, symptom frequency, self-rated medical care, and product availability. Stigmatization was not significantly associated with the total quality of life score. Symptom frequency correlated negatively and moderately with quality of life, whereas ratings of medical care and product availability correlated positively and weakly; less frequent symptoms, higher-rated medical care, and better product availability were each associated with higher quality of life. Because perceived stigmatization was reported by the great majority of respondents but was not associated with the total score, its association with the individual GIQLI domains was examined separately, as its own five-test family. Stigmatization was significantly associated with the emotional domain (ρ = −0.26; unadjusted *p* = 0.010; FDR-adjusted *p* = 0.048), with more frequent perceived stigmatization being associated with poorer emotional quality of life. No other domain reached significance after adjustment: medical treatment (ρ = −0.22; FDR-adjusted *p* = 0.063), physical (ρ = −0.12), symptoms (ρ = −0.11) and social (ρ = −0.09; all FDR-adjusted *p* ≥ 0.348). The domain-level association with emotional functioning is thus stronger than the association with the total score (ρ = −0.17, *p* = 0.085), which is diluted across the four domains that stigmatization does not affect; notably, the association is with emotional rather than social functioning.

### 3.6. Association Between Dietary Adherence (CDAT) and Quality of Life (GIQLI)

Spearman correlation analyses (Table 5) showed that dietary adherence was significantly associated with the total quality-of-life score and every individual domain. Because a lower CDAT score reflects better adherence, the negative correlations indicate that better dietary adherence was associated with higher quality of life throughout: total GIQLI (ρ = −0.50); medical (ρ = −0.51), emotional (ρ = −0.50), symptom (ρ = −0.41), physical (ρ = −0.42), and social (ρ = −0.31) domains (FDR-adjusted *p* < 0.001 for every domain except the social domain, where the FDR-adjusted *p* = 0.004). The social-domain coefficient should not be interpreted substantively: this four-item subscale showed weak internal consistency in the present sample (α = 0.57; Section 3.7), so the corresponding association is reported for completeness only.

### 3.7. Reliability of the Measurement Instruments

Internal consistency in the present sample was good to excellent. Cronbach’s α was 0.95 for the GIQLI total and 0.73 for the CDAT; across the other multi-item GIQLI domains, it ranged from 0.81 (emotional) to 0.92 (symptoms), with the physical domain at 0.84. The four-item social domain was the exception (α = 0.57), and the single-item medical domain did not yield an α. Because unreliability attenuates observed correlations towards zero, the social-domain association reported in Section 3.6 is likely to underestimate the true association; conversely, an α of 0.57 indicates that these four items do not form a coherent scale in this sample, so no substantive domain-specific conclusion should be drawn from that result in either direction.

### 3.8. Sensitivity Analysis for Shared Symptom Content

After excluding the GIQLI symptoms domain, dietary adherence remained significantly associated with quality of life (ρ = −0.51; *p* < 0.001), slightly stronger than the zero-order association (ρ = −0.50). The partial correlation controlling for post-exposure symptom frequency was ρ = −0.46 (*p* < 0.001). The adherence–quality of life association, therefore, persists independently of the symptom content shared by the two instruments and is not an artifact of item overlap.

### 3.9. Factors Independently Associated with Quality of Life and Adherence

In the multiple linear regression predicting total GIQLI (adjusted R^2^ = 0.41; F (8, 92) = 9.79 (*p* < 0.001); all VIF ≤ 1.27), three predictors were independently significant: poorer dietary adherence (higher CDAT: β = −0.36; *p* < 0.001), more frequent post-gluten symptoms (β = −0.25; *p* = 0.004), and higher-rated medical care (β = 0.19; *p* = 0.026); education, product availability, and sex were marginal (*p* = 0.05–0.08). In the logistic regression for inadequate adherence (CDAT ≥ 13; 71 of 101 respondents), more frequent post-gluten symptoms (adjusted OR = 1.52; *p* = 0.024) and shorter time on the diet (adjusted OR = 0.55 per duration category; *p* = 0.017) were the significant predictors. The full model results are shown in Table 6 and Table 7. Because the events-per-variable ratio was low (4.29), the logistic model should be regarded as exploratory: its odds ratios are point estimates with wide confidence intervals, and they identify candidate correlates for confirmatory study rather than quantifying effects with useful precision.

### 3.10. Household Income and Food Expenditure

Household income and average monthly food expenditure, both recorded as ordinal bands, were examined in relation to both outcomes within their own six-test FDR family. Higher household income was associated with better quality of life (ρ = 0.35; unadjusted *p* < 0.001; FDR-adjusted *p* = 0.002), the only association in this family to survive correction. Household income was weakly associated with adherence in the expected direction but did not survive adjustment (ρ = −0.21; unadjusted *p* = 0.039; FDR-adjusted *p* = 0.059), as was average monthly food expenditure (ρ = −0.21; unadjusted *p* = 0.034; FDR-adjusted *p* = 0.059); food expenditure was unrelated to quality of life (ρ = 0.11; *p* = 0.293). The ratio of food expenditure to household income, derived using documented band midpoints, was associated with poorer quality of life before adjustment only (ρ = −0.23; unadjusted *p* = 0.020; FDR-adjusted *p* = 0.059) and was unrelated to adherence (ρ = 0.04; *p* = 0.715).

When household income was added to the multivariable models, it was itself an independent correlate of quality of life (*p* = 0.042), and the model fit improved slightly (adjusted R^2^ 0.41 to 0.43), but the previously reported coefficients were essentially unchanged: dietary adherence moved from β = −0.36 to β = −0.35 (*p* < 0.001 throughout) and education from β = 0.15 to β = 0.14 (*p* = 0.073 to *p* = 0.099). In the logistic model, household income was not significant (*p* = 0.355), and education remained non-significant (*p* = 0.144 to *p* = 0.220). Household income is, therefore, an additional independent correlate of quality of life rather than a confounder of the relationships reported above.

### 3.11. Sensitivity Analyses of the Multivariable Models

Three sensitivity analyses supported the primary models. First, entering education as a categorical rather than a ranked predictor left the pattern of results unchanged: dietary adherence, symptom frequency and self-rated medical care remained the significant independent correlates of the total GIQLI score, and model fit was essentially identical (adjusted R^2^ = 0.41 in both specifications). The category-specific estimates showed that the marginal effect of education under the ranked coding is carried by the vocational group alone (vocational versus higher education B = −14.00, 95% CI: −28.48 to 0.49, and *p* = 0.058; secondary versus higher education B = −3.29, 95% CI: −12.10 to 5.51, and *p* = 0.459), so the ranked coding does not conceal a uniform educational gradient; in the corresponding logistic model, neither contrast was significant (secondary versus higher education adjusted OR = 2.50, 95% CI: 0.72–10.54, and *p* = 0.173; vocational versus higher education adjusted OR = 3.09, 95% CI: 0.33–76.23, and *p* = 0.386).

Second, re-estimating the logistic model with Firth’s penalized likelihood, which corrects small-sample bias in maximum-likelihood odds ratios, left both significant predictors significant and of similar magnitude (symptom frequency adjusted OR = 1.47, 95% CI: 1.05–2.11, and *p* = 0.025; diet duration adjusted OR = 0.59, 95% CI: 0.36–0.92, and *p* = 0.018). Third, a parsimonious model containing only these two predictors, for which the events-per-variable ratio rose to 15, gave adjusted OR = 1.50 (95% CI: 1.09–2.09; *p* = 0.014) for symptom frequency and adjusted OR = 0.53 (95% CI: 0.34–0.80; *p* = 0.004) for diet duration. The low events-per-variable ratio of the primary model, therefore, does not appear to have distorted its conclusions, although the confidence intervals remain wide.

## 4. Discussion

This study assessed health-related quality of life in adults with celiac disease and identified factors associated with their daily functioning and adherence to the gluten-free diet. The results indicate that living with celiac disease is associated with burden on multiple levels, not only physical health, but also emotional, psychological, and social functioning. These findings are consistent with the current literature showing that, despite the high efficacy of the gluten-free diet, patients continue to face difficulties stemming from the chronic nature of the disease, dietary restrictions, and psychosocial burden.

Even while following a gluten-free diet, many respondents experienced difficulties in everyday functioning, particularly eating away from home, the high cost of gluten-free food, and, among the free-text answers given under the “other” option, the fear of accidental gluten ingestion. These observations align with Psylinakis et al. [27], who emphasized that although the gluten-free diet is the foundation of treatment, it can simultaneously limit patients’ comfort and social functioning, with the ubiquity of gluten, the risk of cross-contamination, and social constraints among the principal barriers that lower emotional quality of life. A recent Canadian study framed these difficulties through the concept of food literacy, reporting that adults with celiac disease struggle not only with functional skills such as reading labels and preventing cross-contamination but also with the relational and social dimensions of eating, including social exclusion and mourning the loss of familiar foods [32].

Kamińska et al. [33] found that the emotional and symptom domains were among the lowest-rated areas of quality of life in celiac patients, attributing this to the psychological burden of a chronic disease requiring strict dietary control. This corresponds closely to the present findings, in which the lowest quality of life scores occurred in the emotional, physical and treatment-related domains, which did not differ significantly from one another; a recent meta-analysis likewise reported elevated rates of anxiety and depression among both adults and children with celiac disease [34].

A key result was the relationship between dietary adherence and quality of life: patients with better adherence achieved higher quality of life scores in the total score and in every domain except the social domain, which is not interpreted here because of its weak internal consistency in this sample (α = 0.57). Similar observations were reported by Rustagi et al. [35], who noted that strict dietary control not only reduces symptom frequency but also benefits physical and psychological functioning. It should be noted, however, that not all studies confirm this relationship in adults; the meta-analysis by Guennouni et al. [36] found significant quality-of-life improvement after strict gluten-free dieting mainly in children, without such clear differences among adults.

The cross-sectional design does not permit any statement about direction. Better adherence may support better quality of life, but the reverse is equally plausible: patients with more energy, less emotional distress and more favorable social and financial circumstances may find a demanding, restrictive diet easier to maintain. A reciprocal relationship, in which symptomatic improvement reinforces adherence and sustained adherence maintains that improvement, is also consistent with the present data. Longitudinal designs are required to distinguish among these possibilities.

The sensitivity analyses are informative in this context. Had the association between adherence and quality of life been driven mainly by the symptom-related items that the CDAT and the GIQLI share, excluding the GIQLI symptom subscale should have weakened it appreciably. Instead, the association was marginally stronger without that subscale (ρ = −0.51) than with it (ρ = −0.50) and remained substantial after adjustment for post-exposure symptom frequency (partial ρ = −0.46). Consistent with this, the strongest domain-level associations were observed for the emotional and medical treatment domains rather than for the symptom domain. Together, these findings indicate that dietary adherence relates to how patients feel about and manage their condition, and not merely to the somatic consequences of gluten exposure.

Statistical significance should, nevertheless, be distinguished from clinical relevance. The adherence coefficient of −1.88 GIQLI points per CDAT point implies that a six-point difference in CDAT score—roughly the distance between adequate adherence and clearly insufficient dietary discipline—corresponds to approximately 11 GIQLI points, or about half a standard deviation in this sample, a difference likely to be perceptible to patients. The associations with self-rated medical care (approximately 4 GIQLI points per rating level) and product availability (approximately 7 points) are considerably smaller—approximately 0.17 and 0.31 standard deviations respectively—and their individual-level relevance is correspondingly uncertain. No minimal clinically important difference has been established for the GIQLI in celiac disease, so these comparisons are offered as an aid to interpretation rather than as formal thresholds.

Several mechanisms may plausibly underlie these associations, although the present design cannot test them. Biologically, sustained adherence permits mucosal recovery and resolution of malabsorption, with consequences for fatigue, anemia-related symptoms and micronutrient status that could underlie the physical-domain findings. Behaviorally, adherence requires a demanding repertoire of skills—label reading, control of cross-contamination, and planning meals away from home—and self-efficacy in these skills reduces both actual exposure and anticipatory anxiety, which is consistent with the strong associations observed for the emotional and medical treatment domains. Psychosocially, the diet restructures shared eating, and adherence maintained at the cost of social withdrawal may protect the symptom domain while burdening the emotional one.

A substantial proportion of respondents reported difficulties adhering to the diet, which may stem from the presence of gluten in many foods and the risk of inadvertent exposure. Kowalski et al. [37] showed that even patients confident in their dietary habits often make unintentional errors due to insufficient knowledge or cross-contamination, underscoring the need for continuous patient education and regular dietary monitoring. Consistent with Rahimi et al. [38], who linked adherence difficulties to greater symptom frequency and poorer psychosocial functioning (including higher rates of depression) [39], respondents in the present study who reported more frequent symptoms after gluten intake had lower quality-of-life scores.

The proportion of participants classified as inadequately adherent (70.30%) was higher than might be expected in a group recruited from celiac support communities, and two explanations should be considered. First, the cut-off of 13 points is a screening threshold designed to be sensitive to any risk of gluten exposure rather than a diagnostic criterion of non-adherence; using the more restrictive threshold of 17 points, 26.73% of our respondents would be classified as insufficiently adherent, a figure more in line with CDAT-based studies in Polish and other European adult populations [27,31]. In the Polish CDAT study of Gładyś et al. [31], 52% of 92 adults were classified as insufficiently or poorly adherent at the same threshold of 13 points, and in the Hellenic study of Psylinakis et al. [27], 61.8% of 102 adults scored at or above 13 points and 19.6% above 17 points, compared with 70.30% and 26.73% in the present sample; these studies differ in setting, case mix and recruitment channel, so the comparison is indicative rather than exact, but our estimates are of the same order as those obtained in comparable European adult samples with the same instrument. Second, membership of online support groups may itself be associated with greater symptom burden and greater dietary difficulty, since these are the circumstances that motivate people to seek peer support; this may inflate the estimated burden relative to the general adult celiac population.

Longer time on the gluten-free diet was associated with better adherence in the adjusted model, although the corresponding bivariate association did not survive correction for multiple testing; the two estimates differ because the adjusted model holds symptom frequency and the remaining covariates constant and dichotomizes the outcome at the CDAT cut-off. Because the design is cross-sectional, this pattern cannot be read as evidence that time on the diet improves adherence. Gradual adaptation to living with a chronic disease and the acquisition of practical skills in meal planning and avoidance of accidental exposure are one possible explanation; selective attrition is at least as plausible, since individuals who discontinue strict adherence are less likely to remain active in celiac support communities and, therefore, less likely to have been recruited. Similar temporal patterns have been reported by Kowalski et al. [37], whose patients with longer-standing diagnoses showed greater awareness of dietary principles, and by a large German survey in which negative emotions and dietary difficulties were most pronounced in the first months after diagnosis, and dietary compliance was higher among those diagnosed five to ten years earlier [40]; both comparisons are, like ours, between-person rather than within-person and are open to the same alternative explanation.

A significant positive association was found between the availability of gluten-free products and overall quality of life. Ribeiro et al. [41] similarly identified limited availability and high prices of gluten-free products as major treatment-related difficulties that can impose financial strain on patients and families, potentially undermining adherence and lowering quality of life. A recent Lithuanian study likewise identified product cost as the leading barrier to gluten-free-diet compliance [42]. Education level was also relevant: participants with vocational education showed poorer adherence and reported lower quality of life than those with higher education, consistent with Ribeiro et al. [41], who linked lower education to reduced health awareness, difficulty interpreting dietary recommendations, and limited financial resources. This association should not be overstated. Although participants with vocational education showed poorer adherence in the bivariate comparison, the effect was attenuated to non-significance once symptom frequency and diet duration were included in the multivariable model (adjusted OR = 0.48; 95% CI 0.16–1.21; *p* = 0.144). The bivariate association is, therefore, consistent with the literature cited, but the present data do not demonstrate an independent contribution of education to dietary adherence, and the small size of the vocational subgroup (*n* = 8) further limits what can be concluded. A recent decade-long comparison in the United Kingdom confirmed that gluten-free foods remain limited in availability and substantially more expensive, up to about 180% costlier than their gluten-containing equivalents, even though the relative price gap has narrowed somewhat over time [43]. Food insecurity may compound these barriers: in a large cross-sectional study of adults with celiac disease, adherence to the gluten-free diet partially mediated the relationship between food insecurity and health-related quality of life, with women and lower-income patients reporting poorer quality of life [44]. Consistent with the role of awareness and resources, another study of adults with celiac disease found that understanding dietary requirements, reading nutrition labels, and receiving financial support were each associated with better adherence, while high cost was reported as a barrier by the large majority of patients [45]. Consistent with this, household income was itself associated with quality of life in the present sample (ρ = 0.35; FDR-adjusted *p* = 0.002) and remained an independent correlate when added to the multivariable model, whereas neither income nor food expenditure was significantly associated with dietary adherence after correction. In this sample, economic resources, therefore, appear to bear more directly on how well patients live with the disease than on how strictly they follow the diet.

No significant associations were found between age or sex and either quality of life or adherence. This partly differs from Kamińska et al. [33], who reported that women more often described emotional burden and lower quality of life in selected psychosocial domains; however, in both studies, women constituted the large majority of respondents, which may limit between-sex comparison. Finally, higher-rated medical care was associated with better quality of life, echoing Elwenspoek et al. [46], who emphasized that adequate support from medical staff, access to reliable health education, and dietary consultation meaningfully influence the functioning of patients with celiac disease. This association should be interpreted with particular caution. Both variables are subjective self-reports collected in the same questionnaire, so the observed relationship may partly reflect common-method variance, and the causal direction cannot be established. Patients who are functioning well may evaluate their care more favorably, just as good care may improve functioning. An objective indicator of care quality—for example, documented dietetic follow-up or the frequency of specialist review—would be needed to disentangle these possibilities.

Perceived stigmatization was reported by 90.10% of respondents, yet it was not significantly associated with the total GIQLI score. The domain-level analysis explains why. Stigmatization was significantly associated with the emotional domain (ρ = −0.26; FDR-adjusted *p* = 0.048) but not with the symptom, physical or social domains, so its effect is confined to one of five domains and is correspondingly diluted in a total score dominated by the nineteen symptom items. Notably, the association was with emotional rather than social functioning. What respondents appear to carry from experiences of stigmatization is affective burden—anxiety, frustration, and feeling different—rather than a measurable curtailment of social activity, which is consistent with the observation that the emotional domain was the lowest-scoring domain overall. Two caveats apply: The near-universal endorsement of stigmatization leaves little between-person variance with which associations can be detected, and the GIQLI is a generic gastrointestinal instrument that does not assess the identity- and disclosure-related dimensions of stigma specific to celiac disease; instruments containing dedicated stigma subscales, such as the Celiac Disease Quality of Life measure, would be better suited to testing this relationship directly.

### Limitation

This study has several limitations. First and most important, all of its variables are self-reported: the celiac disease diagnosis, dietary adherence, symptoms after gluten exposure, quality of life, and the ratings of medical care and product availability were obtained exclusively through an anonymous online questionnaire, without serological testing, histological confirmation, medical record review or dietetic assessment. Misclassification of the diagnosis, therefore, cannot be excluded, and socially desirable responding may have biased the adherence and symptom reports; this concern is reinforced by evidence that, even under specialist follow-up, neither symptom resolution nor serological normalization reliably reflects mucosal healing in treated celiac disease [47]. Second, the cross-sectional design precludes causal inference, and reverse or bidirectional relationships between adherence and quality of life are as consistent with these data as a unidirectional one. Third, recruitment through online support communities may bias the sample in two opposing directions: members of such groups may be more engaged and health-literate than the general adult celiac population, which would understate the burden of disease, but people who seek peer support are often those experiencing greater symptom burden, greater dietary difficulty or greater emotional distress, which would overstate it. Because the central descriptive findings of this study concern the proportion with inadequate adherence and the domains scoring lowest, the second mechanism is of particular concern, and none of the prevalence figures reported here should be treated as estimates for the adult celiac population as a whole. Fourth, the sample was predominantly female (84.16%) and highly educated (70.30% with higher education, far above the corresponding proportion in the adult Polish population), which limits generalizability; with only 16 male participants, the absence of a significant sex difference reflects limited statistical power and should not be read as evidence that none exists. Fifth, the education-related differences rest on a vocational education subgroup of only eight participants and should be regarded as preliminary. Sixth, several clinical characteristics known to influence gastrointestinal quality of life were not recorded: time since diagnosis as distinct from time on the diet, coexisting autoimmune disease, persistent gastrointestinal symptoms unrelated to identified gluten exposure, documented nutritional deficiencies, nutritional counselling received after diagnosis, disease severity at diagnosis, and diagnosed psychiatric comorbidity; residual confounding of the reported associations, therefore, cannot be excluded. Psychiatric comorbidity is a particularly consequential omission, given the elevated prevalence of anxiety and depression in celiac disease [34] and the fact that the emotional domain scored lowest in this sample; part of what is interpreted here as disease-related emotional burden may reflect unmeasured psychiatric morbidity. Seventh, the CDAT and the GIQLI symptom domain share symptom-related item content, addressed here by two sensitivity analyses (Section 2.3 and Section 3.8). Eighth, perceived stigmatization, self-rated quality of medical care and gluten-free product availability were each assessed with a single, non-validated item measuring a perception rather than an objective characteristic, and single-item measures have unknown reliability, which attenuates the associations that can be detected; the same caution applies to the medical care association, where predictor and outcome are subjective judgements provided by the same respondent at the same moment. Ninth, the GIQLI social domain showed weak internal consistency (α = 0.57), so no domain-specific conclusion should be drawn from it. Finally, a large number of statistical tests were performed; although *p*-values within each correlation family and within the family of omnibus group comparisons were FDR-adjusted, this controls the expected proportion of false discoveries within a family rather than guaranteeing the validity of any individual result; the regression models were not corrected for multiple testing, and no correction was applied across families. Only the association between dietary adherence and quality of life, which is moderate in magnitude and robust to two independent sensitivity analyses, should be regarded as well supported by these data; the remaining associations are exploratory and require independent confirmation. Future research would benefit from larger, more representative and prospective samples that record the diagnostic modality and the clinical characteristics listed above, combine self-report instruments with objective clinical markers, and include a celiac-specific quality-of-life measure.

## 5. Conclusions

In this sample of internet-recruited adults with celiac disease, quality of life scored lowest in the emotional and physical domains, while the symptom and social domains scored comparatively higher; because the design was cross-sectional and included no comparison group, these differences describe the relative standing of the domains within the sample rather than showing that celiac disease reduces quality of life. The most frequently reported difficulties involved eating away from home, the high cost of the gluten-free diet, and limited product availability; abdominal pain, bloating, fatigue, diarrhea, and headache were the most common symptoms after inadvertent gluten intake. Better adherence to the gluten-free diet was associated with higher quality of life in the total score and in all domains except the social domain, whose weak internal consistency (α = 0.57) precludes interpretation, and longer diet duration was associated with better adherence. Higher quality of life was further associated with milder symptoms, better-rated medical care, greater product availability, and higher household income. Education level appeared related to quality of life in bivariate analysis, whereas age and sex were not; because the vocational-education subgroup was small (*n* = 8) and the association with adherence did not persist after adjustment, this finding is exploratory and needs confirmation. Because the design is cross-sectional, these findings identify correlates of patient functioning rather than causes of it, and this study does not test whether any intervention improves outcomes. The observation that the emotional domain scored lowest, taken together with published evidence of elevated anxiety and depression in this population, suggests that routine screening for psychological distress, access to psychological support, and measures improving the availability of gluten-free food merit evaluation as components of celiac care; this remains a hypothesis for prospective and interventional research rather than a conclusion of the present study.

## Figures and Tables

**Table 1:** Sociodemographic characteristics of the study group (*n* = 101).

Characteristic	Category	*n*	%
Sex	Female	85	84.16
	Male	16	15.84
Education	Higher	71	70.30
	Secondary	22	21.78
	Vocational	8	7.92
Place of residence	City > 500,000	40	39.60
	Rural area	26	25.74
	City 151,000–500,000	16	15.84
	City 51,000–150,000	10	9.90
	City ≤ 50,000	9	8.91
Household size (persons)	1	15	14.85
	2	20	19.80
	3	16	15.84
	4	33	32.67
	5	15	14.85
	6	2	1.98
Only person on GFD in household	Yes	78	77.23
	No	22	21.78

GFD, gluten-free diet. Percentages are column-wise within each characteristic and are calculated for the full sample (*n* = 101). Data on the number of household members following a gluten-free diet were missing for one respondent.

**Table 2:** Social and clinical characteristics of the study group (*n* = 101).

Characteristic	Category	*n*	%
Duration of GFD	<1 year	24	23.76
	1–3 years	31	30.69
	4–10 years	19	18.81
	>10 years	27	26.73
Perceived stigmatization	Yes, often	52	51.49
	Yes, sometimes	39	38.61
	Rarely	7	6.93
	Never	3	2.97
Availability of GF products	Always available	7	6.93
	Usually available	74	73.27
	Rarely available	18	17.82
	None available in the area	2	1.98
Most common symptoms after gluten intake †	Abdominal pain	74	73.27
	Bloating	69	68.32
	Fatigue/weakness	47	46.53
	Diarrhea	45	44.55
	Headache	41	40.59
	Concentration problems	37	36.63

GFD, gluten-free diet; GF, gluten-free. † Respondents could select more than one answer.

**Table 3:** Descriptive statistics of the quantitative variables (*n* = 101).

Variable	M	Me	SD	Sk.	Kurt.	Min.	Max.
Dietary adherence (CDAT)	15.24	14.00	4.40	1.16	3.00	7.00	35.00
Quality of life (GIQLI total)	94.13	97.00	22.80	−0.53	−0.10	31.00	141.00
Symptom domain	2.82	2.95	0.70	−0.80	0.22	0.95	3.95
Emotional domain	2.13	2.20	0.74	0.16	−0.58	0.80	4.00
Physical domain	2.24	2.29	0.79	−0.03	−0.60	0.43	3.86
Social domain	2.98	3.00	0.72	−0.60	−0.35	1.25	4.00
Medical domain	2.30	2.00	1.18	0.00	−0.97	0.00	4.00

M, mean; Me, median; SD, standard deviation; Sk., skewness; Kurt., kurtosis. GIQLI total range: 0–144 (higher = better); CDAT range: 7–35 (lower = better adherence). Domain scores are mean item scores (0–4).

**Table 4:** Spearman correlations of quality of life (GIQLI total) with stigmatization, symptom frequency, medical care, and product availability.

Variable	Spearman ρ	*p* (Unadjusted)	*p* (FDR-Adjusted)
Stigmatization	−0.17	0.085	n/a
Symptom frequency	**−0.34**	<0.001	0.002
Rating of medical care	**0.27**	0.006	0.012
Product availability	**0.28**	0.004	0.010

Both unadjusted and Benjamini–Hochberg FDR-adjusted *p*-values are reported; bold indicates statistical significance after adjustment (*p* < 0.05). The FDR adjustment was applied within the 78-test main correlation family described in Section 2.3; perceived stigmatization was analyzed as an ordinal variable outside that family, its association with the total score is reported unadjusted and is not marked as significant, whereas its five domain-level associations were adjusted within their own five-test family (Section 3.5).

**Table 5:** Spearman correlations between dietary adherence (CDAT) and quality-of-life dimensions (GIQLI).

Quality-of-Life Dimension	Spearman ρ	*p* (Unadjusted)	*p* (FDR-Adjusted)
Total quality of life	**−0.50**	<0.001	<0.001
Symptom domain	**−0.41**	<0.001	<0.001
Emotional domain	**−0.50**	<0.001	<0.001
Medical domain	**−0.51**	<0.001	<0.001
Physical domain	**−0.42**	<0.001	<0.001
Social domain	**−0.31**	0.001	0.004

Values are Spearman’s ρ with both unadjusted and Benjamini–Hochberg FDR-adjusted *p*-values; all correlations remained significant at *p* < 0.05 after adjustment (bold). The social-domain result should be interpreted with caution, as this four-item subscale showed weak internal consistency in the present sample (α = 0.57; Section 3.7).

**Table 6:** Multiple linear regression predicting total GIQLI score (*n* = 101).

Predictor	B [95% CI]	β [95% CI]	*p*
**Dietary adherence (CDAT, per point)**	**−1.88 [−2.75, −1.01]**	**−0.36 [−0.53, −0.19]**	**<0.001**
Education (per level, ranked)	5.60 [−0.54, 11.74]	0.15 [−0.02, 0.32]	0.073
**Symptom frequency**	**−3.95 [−6.58, −1.31]**	**−0.25 [−0.41, −0.08]**	**0.004**
**Medical care rating**	**3.98 [0.50, 7.46]**	**0.19 [0.02, 0.35]**	**0.026**
GF product availability	6.98 [−0.004, 13.97]	0.17 [−0.001, 0.34]	0.050
Diet duration	1.10 [−2.37, 4.57]	0.05 [−0.12, 0.23]	0.532
Age (years)	−0.29 [−0.70, 0.13]	−0.11 [−0.28, 0.05]	0.176
Sex (male vs. female)	9.40 [−1.09, 19.89]	0.15 [−0.02, 0.32]	0.078

B, unstandardized coefficient; β, standardized coefficient; CI, confidence interval. Model adjusted R^2^ = 0.41, F(8, 92) = 9.79, and *p* < 0.001; all VIF ≤ 1.27. Ordinal predictors (education, symptom frequency, medical care rating, product availability, and diet duration) entered as ranked scores; sex coded as female = 0 or male = 1. Bold = *p* < 0.05.

**Table 7:** Logistic regression predicting inadequate dietary adherence (CDAT ≥ 13; *n* = 101).

Predictor	Adjusted OR [95% CI]	*p*
Education (per level, ranked)	0.48 [0.16, 1.21]	0.144
**Symptom frequency**	**1.52 [1.07, 2.23]**	**0.024**
Medical care rating	1.53 [0.93, 2.63]	0.104
GF product availability	0.68 [0.25, 1.79]	0.441
**Diet duration**	**0.55 [0.33, 0.88]**	**0.017**
Age (years)	0.97 [0.91, 1.03]	0.284
Sex (male vs. female)	0.51 [0.12, 2.22]	0.360

OR, odds ratio; CI, confidence interval. Outcome: inadequate adherence (CDAT ≥ 13); 71/101 (70.30%). McFadden pseudo-R^2^ = 0.18 and AIC = 116.3; all VIF ≤ 1.27. Events-per-variable = 4.29 (below the recommended ≥ 10), so odds ratios are imprecise and should be read with caution. Bold = *p* < 0.05.

## Data Availability

The data presented in this study are available on request from the corresponding author. The data are not publicly available due to privacy restrictions.

## Supplementary Materials

The following supporting information can be downloaded at https://www.mdpi.com/article/10.3390/nu18162701/s1. Supplementary Material S1: Author-designed questionnaire: full item wording, response options and analysis coding.
