# Supplementary material for: Quality of Life and Gluten-Free Diet Adherence in Adult Patients with Celiac Disease: A Cross-Sectional Questionnaire Study
Source: Nutrients. 2026 Aug 19;18(16):2701. doi: 10.3390/nu18162701 (PMC13516819; doi:10.3390/nu18162701)
Supplement: Supplementary file 1 [file nutrients-18-02701-s001.zip › nutrients-4481178-supplementary.pdf]

Supplementary Material S1

Author-designed questionnaire: full item wording, response options and analysis coding

Quality of Life and Gluten-Free Diet Adherence in Adult Patients with Celiac Disease: A Cross-Sectional Questionnaire Study

Scope of this document

This file reproduces the complete author-designed questionnaire used in the present study, in the original Polish and in English translation, together with every response option as presented to respondents and the coding applied in the statistical analyses. It is provided so that readers can assess the face and content validity of the instrument and reproduce the analyses.

The questionnaire comprised 15 items and was administered online as a single form; all items were compulsory. It is not a validated psychometric scale: its items were analysed individually rather than as a composite score, and no reliability coefficient is reported for it.

Coding conventions

Ordinal variables were entered into the correlation and regression models as ranked scores in the direction shown below. Multiple-response items were used descriptively only. Variables marked "descriptive only" were not entered into any inferential model.

Questionnaire items

Item 1. Sex

Original (PL): Płeć

Response options (PL): Kobieta · Mężczyzna

Response options (EN): Female · Male

Coding in analysis: Binary; female = 0 (reference), male = 1. Regression covariate.

Item 2. Age

Original (PL): Wiek:

Response options (PL): (odpowiedź otwarta)

Response options (EN): (open response)

Coding in analysis: Continuous, in years. Regression covariate.

Item 3. Place of residence

Original (PL): Miejsce zamieszkania:

Response options (PL): Wieś · Miasto do 50 tys. mieszkańców · Miasto od 51 tys. do 150 tys. mieszkańców · Miasto od 151 tys. do 500 tys. mieszkańców · Miasto powyżej 500 tys. mieszkańców

Response options (EN): Rural · Town (<50,000 residents) · City (51,000–150,000 residents) · City (151,000–500,000 residents) · City (>500,000 residents)

Coding in analysis: Descriptive only (Table 1).

Item 4. Level of education

Original (PL): Poziom wykształcenia:

Response options (PL): podstawowe · gimnazjalne · zawodowe · średnie · wyższe

Response options (EN): Primary · Lower secondary (gimnazjum) · Vocational · Secondary · Higher education

Coding in analysis: Ranked ordinal score: vocational = 1 < secondary = 2 < higher = 3. Regression covariate.

Note: *Five options were offered, but only three occur in the analysed sample.*

Item 5. Number of people in the household

Original (PL): Ilość osób w gospodarstwie domowym

Response options (PL): (odpowiedź otwarta)

Response options (EN): (open response)

Coding in analysis: Count. Descriptive only (Table 1).

Item 6. Number of people in the household following a gluten-free diet

Original (PL): Ilość osób w gospodarstwie domowym stosujących dietę bezglutenową

Response options (PL): (odpowiedź otwarta)

Response options (EN): (open response)

Coding in analysis: Count. Descriptive only. This is the only variable in the data set with a missing value (1 of 101).

Item 7. What is the net monthly income of your household?

Original (PL): Jaki jest miesięczny dochód netto gospodarstwa domowego?

Response options (PL): poniżej 2000 zł · 2000 – 4000 zł · 4001 – 6000 zł · 6001 – 8000 zł · 8001 – 10000 zł · powyżej 10000 zł

Response options (EN): below 2000 PLN · 2000–4000 PLN · 4001–6000 PLN · 6001–8000 PLN · 8001–10,000 PLN · above 10,000 PLN

Coding in analysis: Ranked ordinal score 1–6 in the order shown. Analysed in Section 3.10.

Item 8. Average amount of money spent on food per month

Original (PL): Średnia kwota wydawanych pieniędzy na produkty spożywcze w przeciągu miesiąca

Response options (PL): poniżej 500 zł · 500 – 1000 zł · 1001 – 1500 zł · 1501 – 2000 zł · 2001 – 2500 zł · powyżej 2500 zł

Response options (EN): below 500 PLN · 500–1000 PLN · 1001–1500 PLN · 1501–2000 PLN · 2001–2500 PLN · above 2500 PLN

Coding in analysis: Ranked ordinal score 1–6 in the order shown. Analysed in Section 3.10; band midpoints (with open top bands extrapolated one band-width beyond their floor) were used only for the expenditure-to-income ratio.

Item 9. Have you ever experienced stigmatization or discrimination because of celiac disease (e.g. at work, at school, in restaurants)?

Original (PL): Czy kiedykolwiek odczuwałeś/aś stygmatyzację lub dyskryminację z powodu celiakii (np. w pracy, szkole, restauracjach)?

Response options (PL): Tak, często · Tak, czasami · Rzadko · Nigdy

Response options (EN): Yes, often · Yes, sometimes · Rarely · Never

Coding in analysis: Ranked ordinal score in ascending order of frequency: never = 1, rarely = 2, yes sometimes = 3, yes often = 4.

Item 10. What difficulties do you encounter in everyday life because of celiac disease? (more than one answer may be selected)

Original (PL): Jakie trudności napotykasz w codziennym życiu związane z celiakią? (można zaznaczyć więcej niż jedną odpowiedź)

Response options (PL): Trudności w znalezieniu odpowiednich produktów spożywczych · Trudności w jedzeniu poza domem (restauracje, spotkania towarzyskie) · Koszty diety bezglutenowej · Zrozumienie i wsparcie ze strony rodziny i przyjaciół · Inne

Response options (EN): Difficulty finding suitable gluten-free products · Difficulty eating outside the home (restaurants, social gatherings) · Cost of the gluten-free diet · Understanding and support from family and friends · Other

Coding in analysis: Multiple response; descriptive only. Free-text answers entered under "Other", which included fear of inadvertent gluten exposure, were reviewed descriptively and were not coded into any variable.

Item 11. Do you consider the availability of gluten-free products in your area sufficient?

Original (PL): Czy uważasz, że dostępność produktów bezglutenowych w Twojej okolicy jest wystarczająca?

Response options (PL): Tak, zawsze mam dostęp do produktów bezglutenowych · Zwykle, ale czasami brakuje niektórych produktów · Nie, rzadko mogę znaleźć produkty bezglutenowe · W ogóle nie ma produktów bezglutenowych w mojej okolicy

Response options (EN): Yes, I always have access to gluten-free products · Usually, but some products are occasionally unavailable · No, I can rarely find gluten-free products · There are no gluten-free products at all in my area

Coding in analysis: Ranked ordinal score in ascending order of availability: none available = 1, rarely = 2, usually = 3, always = 4. Regression covariate.

Item 12. What symptoms do you experience after consuming products containing gluten? (more than one answer may be selected)

Original (PL): Jakie objawy odczuwasz po spożyciu produktów zawierających gluten? (można zaznaczyć więcej niż jedną odpowiedź)

Response options (PL): Ból brzucha · Wzdęcia · Biegunka · Zaparcia · Zmęczenie / osłabienie · Bóle głowy · Wysypka skórna · Bóle stawów · Problemy z koncentracją · Nudności / wymioty · Inne · Nie odczuwam żadnych objawów po spożyciu glutenu

Response options (EN): Abdominal pain · Bloating · Diarrhea · Constipation · Fatigue / weakness · Headaches · Skin rash · Joint pain · Concentration problems · Nausea / vomiting · Other · I experience no symptoms after consuming gluten

Coding in analysis: Multiple response; descriptive only (Table 2).

Item 13. How often do you experience the celiac symptoms indicated above after consuming products containing gluten? (if "I experience no symptoms after consuming gluten" was selected, please select "Not applicable")

Original (PL): Jak często odczuwasz wyżej zaznaczone objawy celiakii po spożyciu produktów zawierających gluten? (jeśli zaznaczono „Nie odczuwam żadnych objawów po spożyciu glutenu" proszę o zaznaczenie „Nie dotyczy")

Response options (PL): Nigdy · Rzadko · Czasami · Często · Zawsze · Nie dotyczy

Response options (EN): Never · Rarely · Sometimes · Often · Always · Not applicable

Coding in analysis: Ranked ordinal score: never = 0, rarely = 1, sometimes = 2, often = 3, always = 4, not applicable = 0. Regression covariate, referred to as "symptom frequency" throughout the manuscript.

***Note:** "Not applicable" and "Never" share the lowest code because the "not applicable" option was offered only to respondents who had already indicated, in item 12, that they experience no symptoms after gluten exposure. Note that this item measures the frequency of post-exposure symptoms, not their intensity.*

Item 14. How long have you been following a gluten-free diet?

Original (PL): Od kiedy stosuje Pan/Pani dietę bezglutenową?

Response options (PL): od mniej niż 1 roku · 1–3 lata · 4–10 lat · ponad 10 lat

Response options (EN): less than 1 year · 1–3 years · 4–10 years · more than 10 years

Coding in analysis: Ranked ordinal score 1–4 in the order shown. Regression covariate.

Item 15. How do you rate the quality of medical care in Poland with regard to the diagnosis and treatment of celiac disease?

Original (PL): Jak ocenia Pan/Pani jakość opieki medycznej w Polsce w zakresie diagnozy i leczenia celiakii?

Response options (PL): Bardzo zła · Zła · Przeciętna · Dobra · Bardzo dobra

Response options (EN): Very poor · Poor · Average · Good · Very good

Coding in analysis: Ranked ordinal score 1–5 in ascending order of rating. Regression covariate.
